# Comparative analysis on environmental and economic performance of agricultural cooperatives and smallholder farmers: The case of grape production in Hebei, China

**DOI:** 10.1371/journal.pone.0245981

**Published:** 2021-01-25

**Authors:** Lei Deng, Lei Chen, Jingjie Zhao, Ruimei Wang

**Affiliations:** 1 School of Information, Beijing Wuzi University, Beijing, China; 2 Beijing Municipal Tax Service, State Taxation Administration, Beijing, China; 3 Chinese Academy of Fiscal Sciences, Beijing, China; 4 College of Economics and Management, China Agricultural University, Beijing, China; University of Maryland College Park, UNITED STATES

## Abstract

Agricultural modernization and intensification have been regarded as a significant way to support agricultural development and improve farm income in China. Agricultural cooperatives have played an important role in promoting the modernization and intensification of Chinese agricultural sector. Given the increasing concerns about environmental harm, however, it still remains unclear whether and the extent to which agricultural cooperatives contributes to reducing environmental impacts of agricultural production. Hence, this study performed an environmental evaluation using life cycle assessment for three different organization forms of grape production in Changli County, Hebei Province, China: smallholder farmers, farmer-owned cooperatives and investor-owned firm-led cooperatives. Then the results of life cycle assessment were monetarized and cost benefit analysis was used to evaluate the economic performance of these three organization forms of grape production. The results demonstrate that investor-owned firm-led cooperatives present an overall improvement in environmental and economic performance with the lowest weighted environmental index (integrating all impact categories into a single score), the highest net profit and the highest total net benefit. The results also show a difference in potential improvement in environmental impacts and economic returns between cooperatives and smallholder farmers. Additionally, the production and application of organic and chemical fertilizer and pesticide have been identified as major contributors to total environmental damage.

## Introduction

In recent years, China has witnessed a rapid development of agricultural cooperatives within the context of a wide range of government policies. In recent government reports (S1 Table), it has been emphasized that the development of agricultural cooperatives is an outstanding impetus that promotes the stable and healthy development of agriculture in China. By the end of 2016, the total number of cooperatives hit nearly 2.8 million, of which about 130.3 thousand are farmer-owned (FOC) and 1794 thousand are investor-owned firm-led cooperatives (IOF) [1–3]. Agricultural cooperatives have become the cornerstone of the linkage between smallholder farmers and markets [4] and the main force that promotes China’s agricultural modernization and intensification [1].

In the literature on this subject, agricultural cooperatives have been widely regarded as an effective way to improve farm income [5, 6]. Despite increasing concerns about agricultural pollution (e.g., the degradation of the agro-ecosystems due to the predatory exploitation of land and the aggravation of agricultural nonpoint-source pollution due to excessive use of pesticides and chemical fertilizers) [7] and the growing discussion on the important role of agricultural cooperatives with the decisions of farmers to adopt environmentally friendly inputs and technologies [8], studies on the environmental performance of agricultural cooperatives are rare, and the existing studies focus mainly on the potential effects of agricultural cooperatives on environmentally friendly behavior of farmers. Few studies have focused on the comparison of the environmental impact between agricultural cooperatives and smallholder farmers [9], which means it is still unclear whether and to what extent agricultural cooperatives reduce the negative environmental impact of agricultural production. Thus, a demonstration study was conducted in Changli County, a typical agricultural region in Hebei Province.

Hebei is an important part of the Beijing-Tianjin-Hebei Urban Agglomeration and the coordinated development of the Beijing-Tianjin-Hebei Region. As an important agro-product supplier of Beijing and Tianjin, Hebei is experiencing a rapid shift to a more sustainable and environmentally friendly production pattern in the process of the Beijing-Tianjin-Hebei integration. Changli County is the national modern agriculture demonstration area and national agriculture industrialization demonstration base, implying an advantage in the level of development of agricultural cooperatives in China. The grape and wine industries are the characteristic and leading industries that make significant contributions to creation of employment, generation of rural income and rural economic development in Changli County. Therefore, grape production in Changli County is strongly representative of modern agricultural production, which may provide a better understanding of the environmental and economic performance of agricultural cooperatives and smallholder farmers.

Life cycle assessment (LCA) has been widely used to help systematically understand the potential environmental impact of production systems by quantifying all resource use and associated emissions [10, 11]. The application of LCA in the agricultural sector is increasing, such as cereals (including rice, wheat and maize) [7, 12], fruits [13, 14], livestock products [15], etc. In addition, LCA has allowed comparisons among different production systems such as organic and conventional production systems [16, 17], and exploring the differences in the environmental impact of other factors such as subsidy policies [7] and mixed production systems [18].

Given the wide use of the LCA method, some authors have argued that LCA needs further development to address economic issues [19–21]. Thus, some other analytical techniques have been combined with LCA such as data envelope analysis [22], GIS and spatial analysis [23], artificial intelligence methods [24, 25], optimization methods [26, 27] and cost benefit analysis [28]. Cost benefit analysis (CBA) is a multicriteria approach emphasizing the balance between benefits and costs from both economic and environmental perspectives, and it allows the consideration of multiple stakeholders (e.g., farmers, government and cooperatives), which can offer implications of great significance for policy makers and cooperative managers to improve the environmental and economic performance of agricultural production [29].

This study aims to contribute to the scant literature on the assessment of the environmental impact of Chinese agricultural cooperatives by utilizing the LCA method combined with the CBA method. The purpose of this study is to investigate whether and to what extent agricultural cooperatives reduce the negative environmental impact of agricultural production from a life cycle perspective. To achieve this aim, we evaluated the emission consequences and the relative economic and environmental cost and benefit of grape production in Changli County and compared the performance of cooperatives to smallholder farmers, and the efficiency of cooperatives and smallholder farmers was estimated to quantify environmental consequences of operational inefficiencies using data envelope analysis (DEA). With respect to the previous literature, this study attempts to assess the performance of cooperatives and smallholder farmers separately with the consideration of their effects on farm income and environment. The results may provide new insights for the improvement of the agroecosystem and farm income in grape production and are expected to be of interest to farmers, managers and policy makers in the grape industry.

## Materials and methods

### Description of study case

This study was conducted in Changli County, a typical agricultural county in Hebei Province. The county is situated in the northeast part of Hebei Province, has a population of 0.54 million and covers an area of 1212 km^2^, of which 64% is arable. The rural population is approximately 0.46 million, of which 0.27 million are rural laborers [30]. Changli has a typical continental monsoon climate with an average temperature of 11°C and an annual precipitation of approximately 638.33 mm. The climate is suitable for grape growth and makes Changli the main grape production region for centuries, which reflects an advantage in the development of grape cooperatives in Hebei Province [31].

Besides, the government of Hebei pays close attention to the sustainable and low carbon development of all industries, including agricultural production, and many policy instruments are designed to support environmentally friendly industries. Thus, the grape cooperatives in Changli County inform their members about these policies and encourage them to adopt more environmentally friendly behaviors. For instance, many cooperatives provide their vinegrowers with access to technical training several times a year and with sustainable and environmentally friendly inputs at a lower price. To meet the requirements of local government, the cooperatives often provide information and technical assistance, make possible vertical integration or contract farming and help vinegrowers sell grapes at a higher price (e.g., group purchasing). At the same time, vinegrowers from cooperatives are subject to regulations of cooperatives aimed at reducing the negative externalities of grape production.

These facts make vinegrowers of cooperatives differ from smallholder farmers. Though a stratified random sampling approach was used to select interviewed vinegrowers, these facts may arise from selection bias. Further study is needed to obtain an explanation of the effects of these differences between cooperatives and smallholder farmers on economic and environmental performance.

### Data source

The data used in this study were mainly from field surveys, prior literature and related databases. The field survey was conducted during July-September 2014, which was completed in two phases. In phase 1, a questionnaire was designed for data collection. The questionnaire focused on individual production and sale information, covering inputs, yields and gross income of grape production. During the second phase, vinegrowers from smallholder farms and cooperatives were interviewed using a stratified random sampling approach. For the data collection of smallholder farmers, six villages were randomly selected in Changli County, with five vinegrowers being randomly chosen in each selected village. For the data collection of cooperatives, five and three FOCs and IOFs were randomly selected, with four vinegrowers being randomly chosen in each selected cooperative. The sample size was identified using a simple random sampling method. In this study, the data format is cross-sectional data. The demographic details of these interviewed vinegrowers are shown in Table 1. All the vinegrowers involved were informed of the study objectives before the interview. This study was approved by the ethnic committee of Beijing Wuzi University. The background data were mainly from prior literature and related databases.

**Table 1:** Description details of interviewed vinegrowers (mean value).

	IOF	FOC	SF	Total
Sample size	12	20	30	62
Age	40.63	42.30	44.51	43.05
Education	3.25	3.08	2.96	3.05
Experience in grape cultivation (in years)	9.83	12.60	10.97	11.28

Education means vinegrowers’ maximum education level, defined equal to 1 if the vinegrower has primary education, 2 if middle education, 3 if the vinegrower has Associate degree and 4 if vinegrower has bachelor degree.

### Life cycle assessment

#### Goal and scope definition

**Fig 1:**
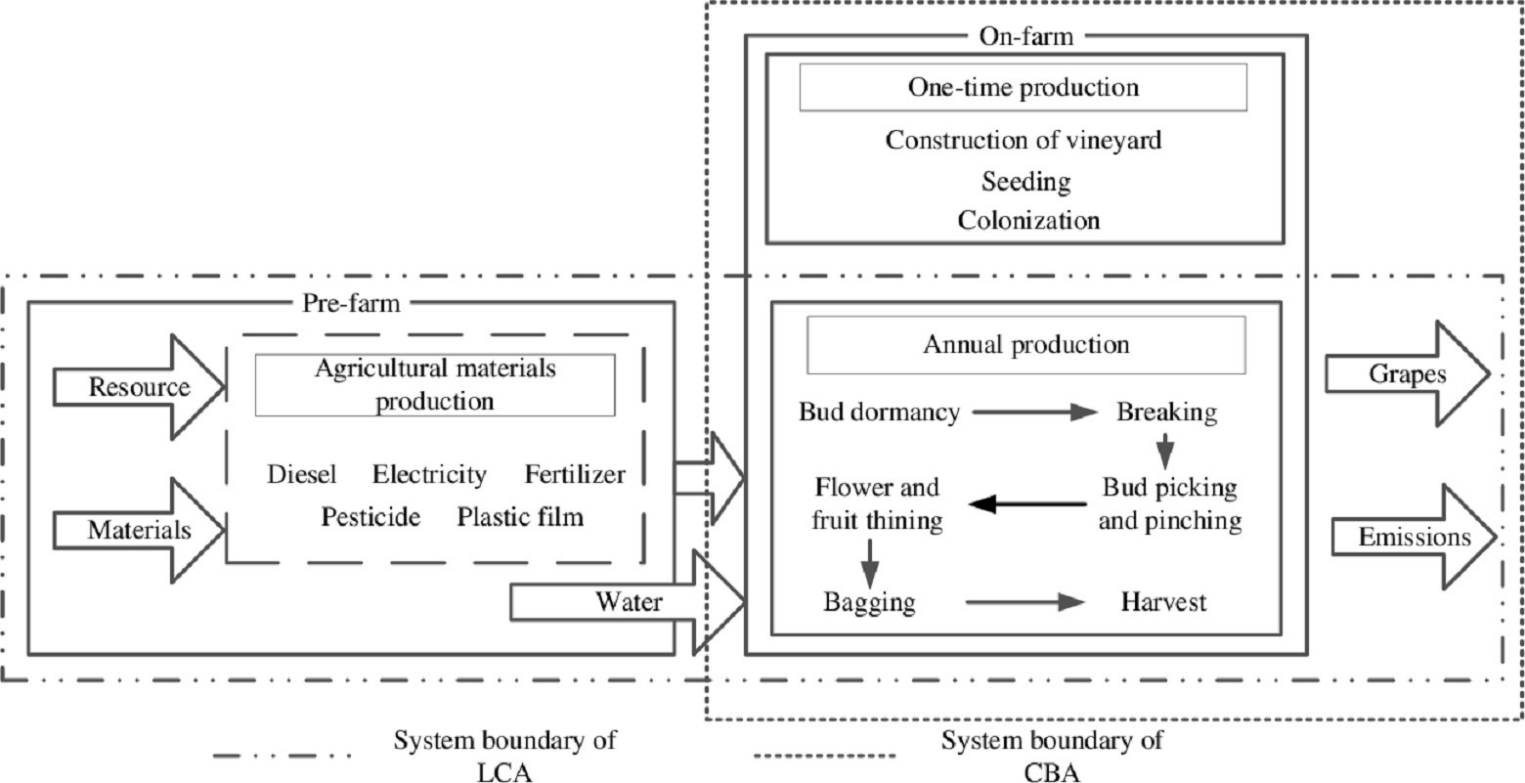
System boundary of LCA and CBA. LCA and CBA represent life cycle assessment and cost benefit analysis, respectively.

The goal and scope definition of an LCA describes the framework of the study regarding system boundary and functional unit. The cradle-to-gate approach is used to set the system boundary, which means this study focuses mainly on the pre-farm stage (e.g., production of raw materials, including electricity, diesel, pesticide, organic and chemical fertilizer and plastic film), on-farm stage (i.e., production of raw materials) and grape cultivation) (Fig 1). The post-farm stage (distribution, processing, transportation and consumption of grapes) was excluded. Due to the lack of data, the transportation of raw materials was ignored. The functional units (FUs) were 1 ton of grapes produced and the yield of the 1-hectare vineyard.

#### Life cycle inventory

The inventory analysis quantified all the resources used and emissions released related to the defined functional unit. The background data were obtained from prior literature and used to modify the data we used for Chinese conditions, as research conducted in China was preferred. Specifically, data sources relating to the inputs to grape cultivation included Hong et al. [32] and Liang [33] regarding organic and chemical fertilizer production and Hu et al. [34] and Leng et al. [35] regarding electricity generation. The data on pesticide production [36], diesel production [33, 34], and plastic film production [37] are taken from the references. Despite their considerable impact on the environmental performance of grape production, the production of trellises, farm machinery and vehicles were ignored in our study based on the facts that the effects of agricultural policies on the production of these materials are negligible and may be beyond the scope of our study since we focus on the difference in resource use between cooperatives and smallholder farmers.

At the on-farm stage, following Brentrup et al. [38] and Zhang et al. [39], the direct emissions of ammonia, nitrous oxide and NO_x_ were 14.25%, 1% and 0.1% of the nitrogen fertilizer used for grapes, respectively. Nitrite leaching from the application of nitrogen fertilizer was estimated as 30% nitrogen input [40]. Additionally, the induced emissions from nitrate losses were considered to be 1% for ammonia-N [38]. According to Wang et al. [41], phosphorus loss through run-off, soil erosion and leaching was calculated as 2% of inputs from phosphorus sources. Meanwhile, the CO_2_ emitted by human labor was also considered in this study, and its emission factor was 0.7 kg CO_2_ eq/man-h according to Nguyen and Hermansen [42].

Heavy metal (Cd, Pb, Cu, Zn) losses from the application of organic and chemical fertilizers and irrigation were estimated according to Liang et al. [7]. The emissions released by utilization of plastic film were calculated according to Shang et al. [37]. The pesticide losses were estimated following a standard residue rate of 10% to air, 1% to freshwater and 43% to soil per unit weight of pesticides [16].

#### Life cycle impact assessment

Impact assessment is a further interpretation of the life cycle inventory data, including characterization, normalization and weighting. Since our purpose is to assess the impacts of grape production, the CML method, which groups life cycle inventory results in midpoint (problem-oriented) impact categories with low uncertainties, was chosen to calculate the 7 relevant impact categories, covering acidification (AC), eutrophication (EP), freshwater aquatic ecotoxicity (FAE), global warming (GW) over 100 years, human toxicity (HT), photochemical oxidation (PO), and terrestrial ecotoxicity (TE). The normalization factors of HT, FAE and TE are based on a global per capita reference for the year 2000 following Sleeswijk et al. [43], while the rest of the parameters are based on a Chinese per capita reference for the year 2010 [7]. The factors established by Liang et al. [7] were used to calculate the weighting index (WI) of different environmental impacts.

### Cost benefit analysis

As pointed out by Wang et al. [41], agricultural cooperatives facilitate the access to large markets for farmers by providing sale contracts. This practice may restrict their choice of agricultural production inputs. In these contracts, the buyers may require size, supplying amount, as well as fertilizer and pesticide residue for agricultural products, or even the certification of green or organic products. In return, farmers may sell their products at a stable and relatively higher price. To take these economic conditions into consideration, a cost benefit analysis was conducted. Additionally, the same FU (1 ton of grapes) was used to maintain consistency between LCA and CBA. The system boundary of the CBA is shown in Fig 1.

#### Economic cost and benefit

The cost associated with each input of grape production is shown in Table 2. The cost of the vineyard was calculated by dividing the sum of its construction cost and maintenance cost by its average lifetime (25 years). The other inputs included grape losses, sales commissions and packaging. Inspired by the work of Deng et al. [31], economic cost included operating cost and fixed cost: the former was estimated based on the cost of pesticides, organic and chemical fertilizer, labor, plastic film, water and energy; the latter consisted of vineyard and farm machinery cost. The economic benefit was represented by net profit, which was equal to the difference between gross return and total economic cost.

**Table 2:** Inputs and outputs per ha of grape production in Hebei.

	IOF (ha^-1^)		FOC (ha^-1^)		SF (ha^-1^)	
	Amount	Cost / benefit (CNY)	Amount	Cost / benefit (CNY)	Amount	Cost / benefit (CNY)
Irrigation water (m^3^)	364.32	1726.87	425.36	2016.22	415.34	1968.70
N (kg)	182.44	12973.90	279.32	14969.79	296.64	15360.00
P (kg)	167.24		174.58		185.40	
K (kg)	276.70		268.85		259.56	
Electricity (kwh)	667.63	334.82	890.18	446.42	749.04	375.64
Organic fertilizer (kg)	11511.93	6649.59	10997.77	6352.59	10179.61	5880.00
Plastic film (kg)	1018.27	5099.80	1347.66	6749.51	1470.56	7365.00
Diesel (liter)	185.50	1328.18	204.41	1463.59	135.41	969.56
Labor (day)	828.75	40339.65	881.25	42895.11	818.79	39855.00
Pesticide (kg)	2.47	1104.59	4.33	1937.45	5.45	2440.00
Yield (kg)	27000.00		29400.00		26948.28	
Vineyard		45856.50		46580.55		24135.00
Farm machinery		3302.58		2433.48		1738.20
Other inputs		4387.50		5062.50		3375.00
Total economic cost		123103.99		130907.21		103462.11
Gross return		210062.50		219308.30		157937.80
Net profit		86958.51		88401.09		54475.69

IOF, FOC and SF represent investor-owned firm-led cooperatives, farmer-owned cooperatives and smallholder farmers, respectively.

#### Environmental cost

There are three major environmental costs generated in grape production in Changli, namely, environmental pollution, ecosystem losses and human damage [44]. As reported by Woon and Lo [45], air emissions were the most important contributors to environmental impact. Thus, we chose the economic cost of air emissions as the indicator of environmental pollution, which was equal to the sum of the amount of emission multiplied by its price. Resource consumption cost, according to Wang et al. [46], was regarded as the indicator of ecosystem losses and was equal to the amount of resource consumption multiplied by resource price. The estimation of environmental pollution and ecosystem losses was based on the following equation:

$${EC}_{P}=\sum P_{i}*A_{i}$$ (1)

where *EC*_*P*_ is the environmental cost in terms of environmental pollution or ecosystem losses; *P*_*i*_ represents the price of the *i*^th^ pollutant emitted or resource consumed; *A*_*i*_ represents the amount of *i*^th^ pollutant emitted or resource consumed.

Additionally, human damage was represented by human capital loss. Following Huijbregts et al. [47] and Liang et al. [7], the Human Capital Approach was used to quantify human capital loss, Disability Adjusted Life Years was used as the indicator of human health impacts, and the endpoint damage factors taken from the Recipe method were adopted to calculate the environmental cost following two equations:

$$D_{j}=f_{j}*A_{j}$$ (2)

$${EC}_{H}=NI*\sum D_{j}$$ (3)

where *D*_*j*_ represents the Disability Adjusted Life Years from the *j*^th^ pollutant; *f*_*j*_ represents the damage factor of the *j*^th^ pollutant; *A*_*j*_ represents the amount of *j*^th^ pollutant; *EC*_*H*_ is the environmental cost in terms of human damage; and *NI* represents the per capita net income of China in 2014 (20566.2 CNY).

Information about the cost of the included environmental impacts was taken from the references [7, 45, 46, 48].

## Results and discussion

### Life cycle assessment results

The life cycle impact assessment results for grape production are shown in Table 3. Taking 1 ton of grapes as FU, the environmental impacts of different organizational forms vary, reflecting different resource inputs. The HT and TE of IOF are the highest among the three organization forms, due mainly to the heavy metal emissions to soil from the use of organic and K fertilizer. Additionally, NH_3_ and NO_2_ released by the application of organic and N fertilizer make great contributions to the highest GW and AC of FOC. The EP, FAE and PO of SF are higher than those of IOF and FOC, which results from the use of organic fertilizer, plastic film and pesticides. The normalization result suggests that FAE is the most important environmental impact for IOF, FOC and SF. Taking SF as an example, this indicator was caused mainly by copper to soil (38.4%), zinc to soil (9.35%) from organic and chemical fertilizers, and pesticide emissions to soil (27.21%), water (12.09%) and air (9.38%), like the results shown in Ferrari et al. [49], implying that the use of pesticides may be the most important source of agricultural pollution in grape production. The WI suggests the best performance in the overall environmental impact of IOF, followed by FOC, possibly implying an environmental improvement from smallholder farmers to agricultural cooperatives, with the WI decreasing by 12.28% compared to IOF and 6% to FOC.

**Table 3:** Impact assessment results per ton / ha of grape production.

Impact category	unit	Cooperatives						Smallholder farmers		
		IOF			FOC					
		EI	NV	WI	EI	NV	WI	EI	NV	WI
AC	kg SO_2_eq/ton	17.466	0.606	0.085	17.471	0.607	0.085	17.378	0.603	0.084
	kg SO_2_eq/ha	478.043	16.599	2.324	490.718	17.039	2.385	462.745	16.068	2.25
EP	kg PO_4_eq/ton	4.906	1.291	0.155	5.055	1.33	0.16	5.092	1.34	0.161
	kg PO_4_eq/ha	135.273	35.598	4.272	141.247	37.17	4.46	135.581	35.679	4.282
FAE	kg 1,4-DBeq/ton	24.026	4.974	0.696	26.451	5.476	0.767	28.86	5.975	0.837
	kg 1,4-DBeq/ha	615.901	127.516	17.852	723.368	149.766	20.967	764.638	158.31	22.163
GW	kg CO_2_eq/ton	1225.926	0.156	0.019	1250.237	0.159	0.019	1210.651	0.154	0.018
	kg CO_2_eq/ha	29985.565	3.813	0.458	32624.087	4.149	0.498	30918.863	3.932	0.472
HT	kg 1,4-DBeq/ton	42.177	0.214	0.03	40.525	0.205	0.029	39.965	0.203	0.028
	kg 1,4-DBeq/ha	1166.116	5.913	0.828	1139.929	5.78	0.809	1064.347	5.397	0.756
PO	kg C_2_H_4_eq/ton	0.091	0.004	0	0.096	0.004	0	0.099	0.004	0
	kg C_2_H_4_eq/ha	2.194	0.092	0.008	2.57	0.107	0.009	2.583	0.108	0.009
TE	kg 1,4-DBeq/ton	2.25	0.368	0.033	2.17	0.355	0.032	2.171	0.355	0.032
	kg 1,4-DBeq/ha	61.117	10.003	0.9	60.971	9.979	0.898	57.851	9.468	0.852
WI	ton^-1^			1.018			1.091			1.161
	ha^-1^			26.641			30.027			30.783

EI, NV and WI represent the environmental impact, normalization values and weighting index, respectively.

Taking 1 ha as FU, the results were similar to the case of 1 ton of grapes. HT and TE are also the highest environmental impact indicators of IOF. Compared to the other organizational forms, AC, EP and GW of FOC are greater. The rest of the impact categories of SF are more significant than IOF and FOC. According to the normalization results, the most significant contribution to total damage is made by FAE. There is an obvious overall improvement in the environmental performance of IOF and FOC compared to SF, with the WI decreasing by 13.45% and 2.46%, respectively.

### Cost benefit analysis results

The most important contributors to total cost were the cost of vineyard, labor and chemical fertilizer, which, taking IOF as an example, accounted for 37.25%, 32.77% and 10.54% of the total cost, respectively. Compared to SF, the vineyard cost of IOF and FOC was much higher, attributed to the high level of skill and management and a better growing environment to meet the requirements of high grape quality and low residues [31]. Additionally, the highest cost was recorded in relation to the operating cost (accounting for 60.07%, 62.56% and 74.99% of the total cost in IOF, FOC and SF, respectively), suggesting the great importance of productivity improvement for increasing farm income. In terms of net profit, IOF presented the best performance, followed by FOC. This result demonstrated that cooperatives were more beneficial from an economic perspective.

The environmental costs were quantified and expressed in CNY per ton of grape production. The results showed a similar ranking of organizational forms with the LCA results. In terms of ecosystem loss and environmental pollution, IOF had the best performance, increasing 2.61% and 2.58% in the case of FOC and 4.09% and 2.81% of SF, respectively. There is a reduction, however, in human damage cost in the case of SF compared to IOF and FOC. SF showed an environmental savings in human damage cost of 0.72 CNY/t and 1.35 CNY/t compared with IOF and FOC, respectively.

Taking economic and environmental performance together, IOF presented the highest total net benefit, followed by FOC and then SF. Assuming a smallholder farmer to be encouraged to join cooperatives, the total benefit will increase in a range of 985.79 CNY/t for FOC to 1207.53 CNY/t for IOF, which implies that cooperatives are more sustainable and beneficial than smallholder farmers. The cost and benefit of grape production are shown in Table 4.

**Table 4:** Cost and benefit of grape production (CNY/t).

	Cooperatives		Smallholder farmers
	IOF	FOC	
**Economic**			
Gross return	7780.09	7459.47	5860.77
Operational cost	-2738.70	-2785.48	-2879.18
Fixed cost	-1820.71	-1667.14	-960.11
Net profit	3220.69	3006.84	2021.49
**Environmental**			
Ecosystem loss	-71.07	-72.93	-73.98
Environmental pollution	-205.08	-210.37	-211.02
Human damage	-59.39	-60.02	-58.67
Total environmental benefit	-338.47	-346.36	-346.8
**Total cost**	-4897.88	-4798.98	-4186.09
**Total net benefit**	2882.22	2660.48	1674.69

Total net benefit is equal to the sum of all items listed in this table (except for net profit).

### Potential improvement in environmental impacts and economic returns of grape production

The results of LCA and CBA prove that the cooperatives have an overall effect on reducing environmental impacts and a higher economic return. To quantify potential environmental consequences of operational inefficiencies in grape production, DEA is used to measure the efficiency of vinegrowers (S2 File). The results can be observed in Table 5.

**Table 5:** Efficiency scores of cooperatives and smallholder farmers.

	Cooperatives		Smallholder farmers
	IOF	FOC	
Average	0.92	0.77	0.66
Standard deviation	0.51	0.28	0.39
Minimum	0.58	0.43	0.32
Maximum	1.34	1.13	1.21
Number of inefficient vinegrowers	5	12	22

According to the target projections computed through DEA, the LCI data of inefficient vinegrowers can be modified. As a result, new environmental impacts assessment results are obtained, representing the potential improvements in environmental impacts of these inefficient vinegrowers. Table 6 shows the average reduction percentages in operational inputs and environmental impacts.

**Table 6:** Average reduction in operational inputs and environmental impacts (%).

Operational inputs		Water	N	P	K	Electricity	Organic fertilizer	Plastic film	Diesel	Labor	Pesticide
**Cooperatives**	**IOF**	13.36	24.61	24.08	22.75	21.23	9.09	24.02	22.54	16.25	16.82
	**FOC**	16.72	42.29	39.87	40.24	39.48	13.07	38.77	35.01	26.58	34.02
**Smallholder farmers**		25.81	44.79	42.94	43.40	47.23	13.59	37.78	33.47	29.11	28.34
Environmental impacts		AC	EP	FAE	GW	HT	PO	TE	WI		
**Cooperatives**	**IOF**	11.24	12.44	11.70	15.73	11.45	18.07	9.98	11.77		
	**FOC**	18.94	21.57	22.27	26.49	18.48	30.52	15.92	21.68		
**Smallholder farmers**		20.39	23.37	21.07	28.35	19.68	31.67	16.35	21.30		

As shown in Table 5, about 62.9% of the assessed vinegrowers in Changli County are deemed inefficient. Specifically, best performer of IOF presents an efficiency of 1.34, higher than that of SF (1.21) and FOC (1.13). On the other hand, vinegrowers of SF shows a lowest average efficiency (0.66) compared to the IOF (0.96) and FOC (0.77), implying a relative high potential in inputs reduction and environmental performance improvement if all inefficient SF vinegrowers follow the optimal operate conditions. In this regard, operational inputs reduction of SF ranged from 13.59% (Organic fertilizer) to 47.23% (Electricity). Taking environmental impacts into consideration, the potential for minimizing the environmental impacts of SF and FOC is higher than that of IOF, which ranges from 16.35% to 28.35% and 15.92% to 26.49%, respectively. In regard to the overall environmental impact (represented by WI), the environmental minimization is potentially high for vinegrowers of FOC. For IOF vinegrowers, however, results suggest little space for reduction in operational inputs and environmental impacts due to their improved operational performance.

The significant average reduction in operational inputs showed in Table 7 also implies an important economic savings in grape production. In other words, a total saving of 12261.49 CNY/ha, 22639.77CNY/ha, 23599.62CNY/ha can be achieved (17.63%, 29.47%, 31.80% reduction in operational cost) by converting inefficient vinegrowers to efficient ones for IOF, FOC and SF, respectively.

**Table 7:** Economic savings for different operational inputs (CNY/ha).

	Cooperatives		Smallholder farmers
	IOF	FOC	
Irrigation water	230.75	337.17	508.03
Chemical fertilizer	3089.19	6107.57	6714.06
Electricity	71.07	176.23	177.41
Organic fertilizer	604.45	830.12	799.03
Plastic film	1224.72	2617.10	2782.57
Diesel	299.34	512.38	324.50
Labor	6556.20	11400.17	11602.45
Pesticide	185.76	659.04	691.56

According to the results of CBA, labor cost is the highest operational cost. However, a significant reduction in labor cost can be achieved if the inefficient vinegrowers operate under the efficient conditions, implying more skilled labors should be employed to increase the productivity and reduce the number of workers (therefore reduce the total cost of labor power) without reduction of yield level.

### Main contributors to environmental impact and economic cost in grape production

**Fig 2:**
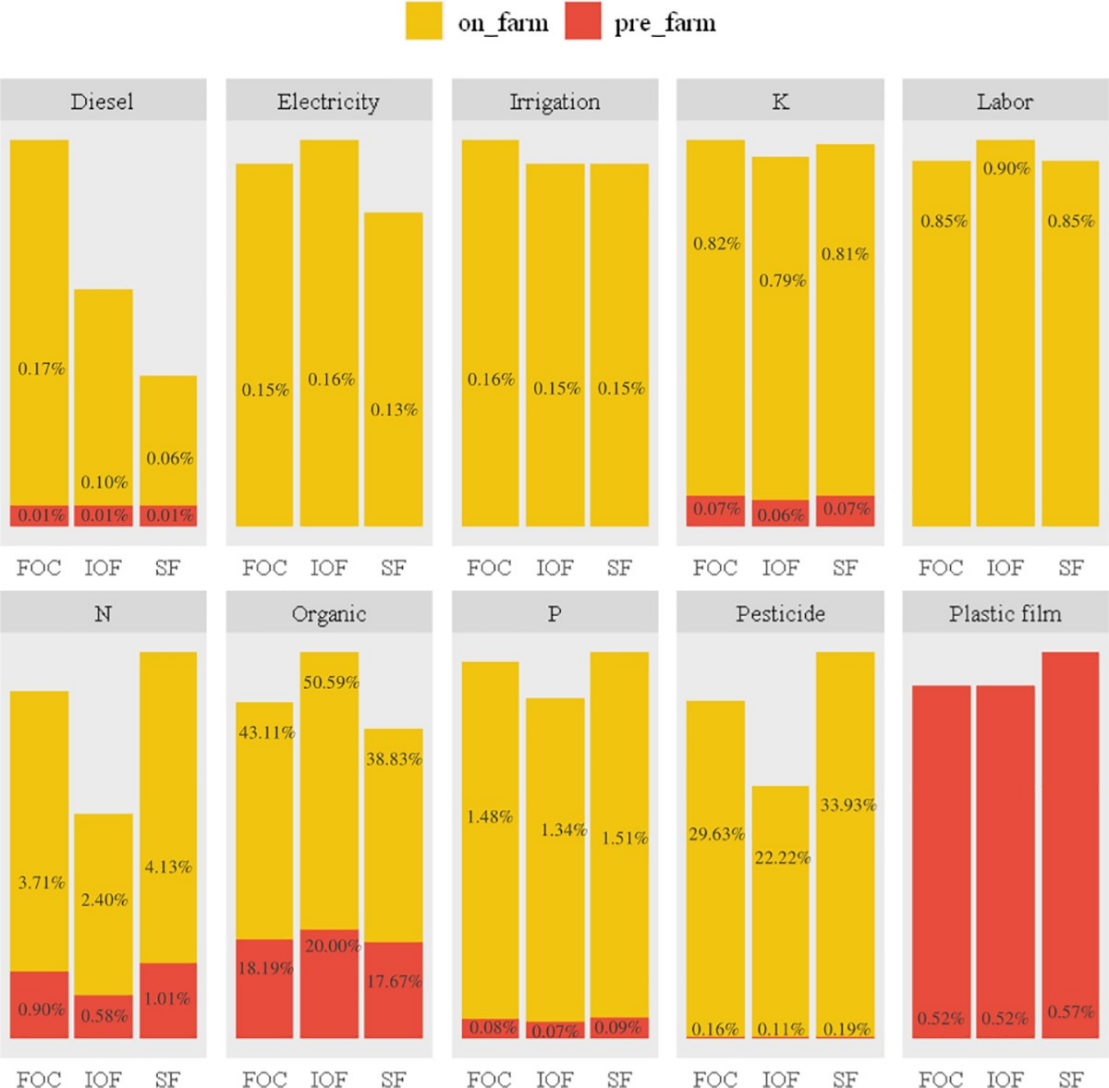
Contributions of different inputs (in %) to WI (per 1 ton grape). IOF, FOC and SF represent investor-owned firm-led cooperatives, farmer-owned cooperatives and smallholder farmers, respectively. WI represents weighting index.

**Fig 3:**
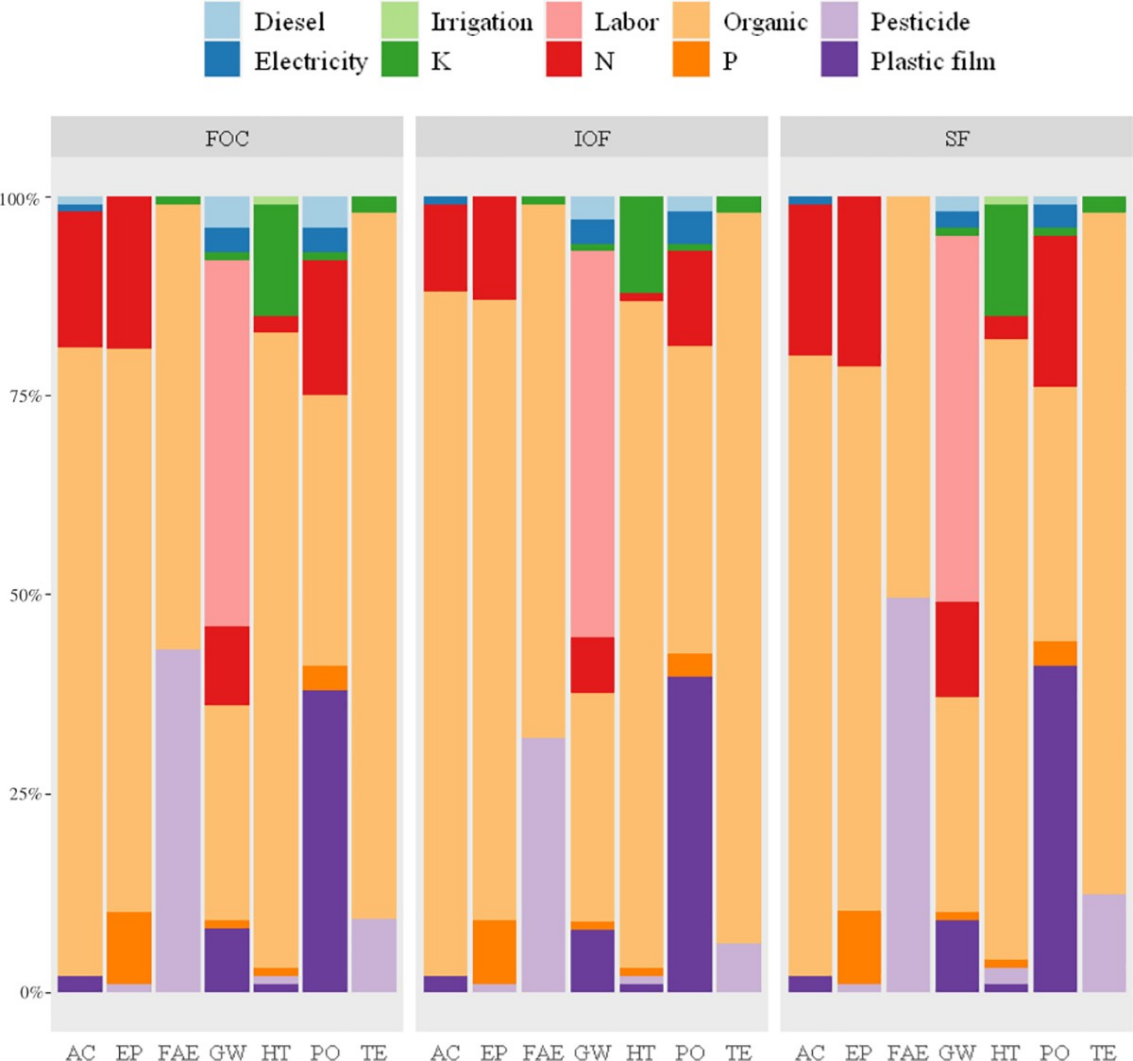
Contributions of different inputs (in %) to environmental impact categories (per 1 ton grape). IOF, FOC and SF represent investor-owned firm-led cooperatives, farmer-owned cooperatives and smallholder farmers, respectively.

The application and production of organic fertilizer were clearly identified as the major contributors to all environmental impacts, except for GW and PO (Figs 2 and 3). The hot spot of PO was the production and use of plastic film. Additionally, the production and use of pesticides and chemical fertilizers also made great contributions to the environmental impacts. Similarly, Mohseni et al. [40] pointed out that the application of organic fertilizer makes the greatest contribution to GW, FAE and TE in grape production in Arak County. Organic fertilizer use in raisin production in Iran is also indicated as a hotspot in GW, EP and TE, while its production is the major contributor to FAE, PO and HT [10]. Based on the results, the environmental sustainability of grape production in Hebei has been reduced since the indiscriminate use of organic fertilizer and pesticides.

**Fig 4:**
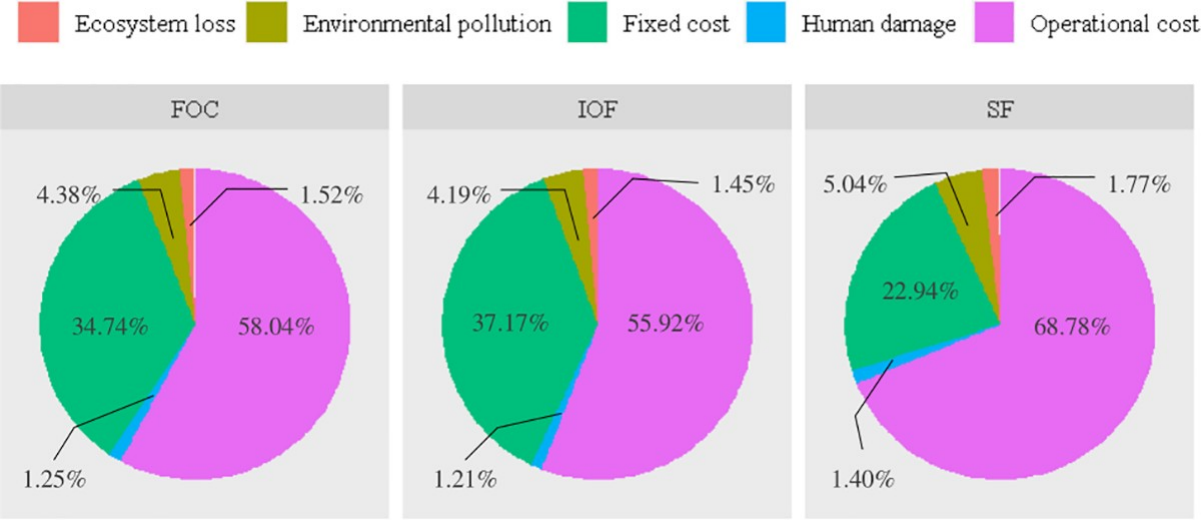
Structure of economic and environmental costs (per 1 ton grape). Fixed cost consists of vineyard and farm machinery cost, operational cost consists of pesticide, organic and chemical fertilizer, labor, plastic film, water and energy cost.

**Fig 5:**
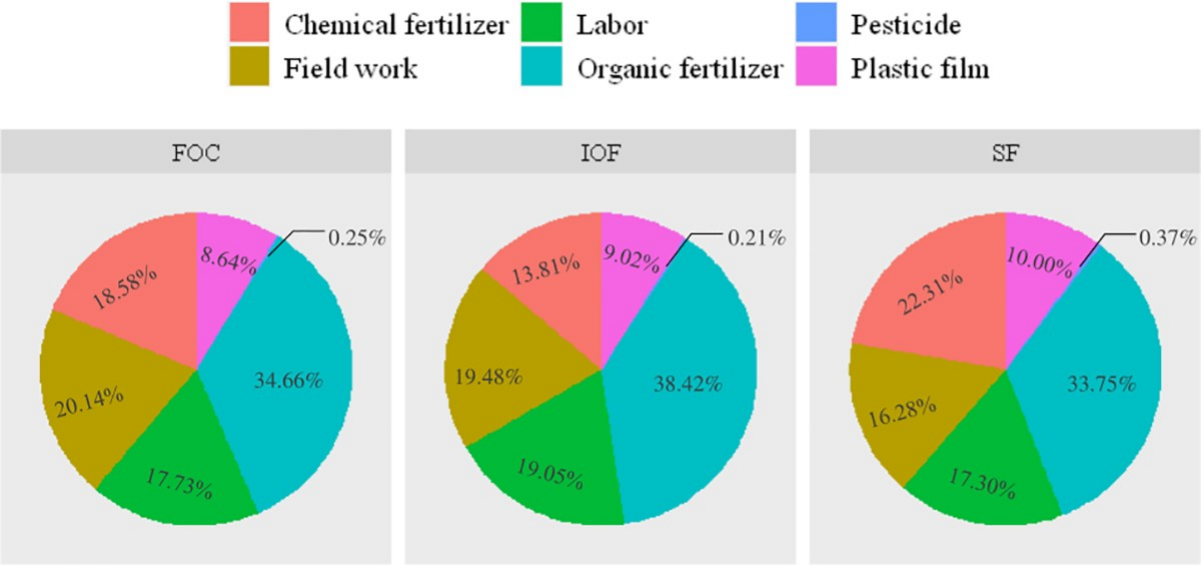
Contributions of different inputs (in %) to the total environmental cost (per 1 ton grape). Fieldwork consists of energy and resource use at on-farm stage (i.e. diesel, electricity and irrigation water).

In terms of environmental cost, for simplicity, energy and resource use at the on-farm stage (i.e., diesel, electricity and irrigation water) were grouped and expressed in terms of fieldwork. Information on the structure of economic and environmental costs of grape production is shown in Figs 4 and 5. The contribution of each input to environmental cost is shown in Table 8.

**Table 8:** Contributions of input to environmental cost (CNY/t).

	Cooperatives						Smallholder farmers		
	IOF			FOC					
	EL	EP	HD	EL	EP	HD	EL	EP	HD
Fieldwork	48.69	15.03	2.23	48.44	18.44	2.88	43.66	11.14	1.65
Chemical fertilizer	11.46	29.72	5.57	14.02	41.99	8.34	19.38	48.77	9.22
Organic fertilizer	7.66	91.51	30.88	7.09	84.38	28.57	6.9	82.34	27.79
Pesticide	0.48	0.16	0.06	0.53	0.23	0.09	0.88	0.29	0.11
Labor	0	47.25	17.24	0	44.77	16.65	0	43.95	16.04
Plastic film	2.78	24.35	3.4	2.85	23.61	3.48	3.16	27.66	3.86
Total cost	71.07	208.01	59.39	72.93	213.43	60.02	73.98	214.15	58.67

EL, EP and HD represents Ecosystem loss, Environmental pollution and Human damage, respectively.

The largest cost, obviously, was recorded in relation to environmental pollution whose major contributors were organic fertilizer and labor power. As reported by Hedayati et al. [50], agricultural production systems play an important role in reducing air emissions. These results suggest more attention should be paid to emissions management, efficiency improvement and sustainable and renewable energy use. Considering ecosystem loss cost, the cost of fieldwork was the highest, followed by chemical fertilizer. This was a result of diesel and coal consumption. Finally, the human damage cost was attributed mainly to organic fertilizer and labor power. These results were in line with the study of Ferrari et al. [49] and Liang et al. [7].

Taking the environmental cost together, the results showed that the major contributors to the total environmental cost of IOF, FOC and SF were different (Fig 5). The application and production of organic fertilizer were identified as major contributors to the total environmental cost. Policy makers and managers should pay more attention to soil management and improve the amount and type of organic fertilizer used for grape production. The following contributors were fieldwork, labor power and chemical fertilizer. These results showed that the difference in the amount of inputs among IOF, FOC and SF should be considered when policy instruments are designed.

However, diesel combustion, which is identified as a hot spot of grape production in other countries [51], made a relatively low contribution to the total environmental impact. For instance, Vázquez-Rowe et al. [52] argued that diesel and chemical fertilizer use were hotspots in GW. In addition, Point et al. [53] proposed similar results in their study on grape production in Canada. In our study, however, GW was mainly caused by GHG emissions from labor power. As a labor-intensive industry, the requirement for manual operations is high at each stage (e.g., from planting to harvesting), which is equal to 750–840 man-d per hectare of open field grape production and approximately 1125 man-d per hectare of protected grape production. Trellises, which have been identified as environmental hotspots in previous studies [53, 54], were not included in our study. The relevant literatures are listed in Table 9.

**Table 9:** Main contributors to environmental impact categories of previous studies.

Literature	LCIA method	Main contributors in grape production	Final products
Villanueva-Rey et al. [51]	CML	Diesel, trellis, pesticide	Wine
Behzad et al. [10]	CML	Organic fertilizer, pesticide	Raisin
Point et al. [53]	CML	Fertilizer (organic and chemical), diesel, trellis	Wine
Meneses et al. [54]	ReCipe	Fertilizer (organic and chemical), pesticide	Red wine
Ferrari et al. [49]	IMPACT	Fertilizer (organic and chemical), diesel	Red wine
Mohseni et al. [40]	CML	Fertilizer (organic and chemical), diesel	Grape

Some of the studies listed in this table focused on both stages of vineyard and post-vineyard. Therefore, we only compared the results related to the emissions from vineyard stage. Meanwhile, considering the difference in environmental impact categories between different LCIA methods, we also only focused on the impact categories discussed in this study.

Meanwhile, the energy consumption from transportation of raw materials (e.g., organic fertilizer, pesticide) was excluded in our study. As a result, the contribution of organic fertilizer may be underestimated. Vinegrowers have to purchase organic fertilizer from markets far from the vineyards due to its shortage [16]. In addition, considering that the grape production system is dominated by small-scale and dispersed vinegrowers [31], the energy consumption of organic fertilizer in the transportation stage may be higher than that of other inputs and may represent a significant contribution to the relative impact categories.

Considering economic performance, the greatest contribution to economic cost was made by labor power, followed by chemical fertilizers, in line with some previous studies [55]. Hebei Province is a typical agricultural region in China, where many of China’s agro-products are produced. The excessive use of chemical fertilizers is the primary source of nonpoint source pollution in Hebei Province. Thus, the proper amount and type of chemical fertilizers used for grape production may be an effective way to improve the environmental and economic performance of grape production in Changli County.

### Agricultural cooperatives and environmental performance

Agricultural cooperatives play an important role in improving agricultural sustainability in China through helping farmers to adopt eco-friendly technologies and access environmentally friendly inputs with lower price, promoting organic agricultural production and enhancing sustainable use of material inputs and natural resources [2]. Our results suggest that the agricultural cooperatives have led to improvement in overall environmental and economic performance. This is consistent with a raft of previous studies. For example, Ma et al. [56] reported that cooperatives encouraged its members to invest in organic soil amendments and eco-friendly fertilizers. Ji et al. [2] also pointed out that joining cooperatives has a significant and positive effect on farmers’ willingness to adopt safe production behaviors. Cai et al. [57] also documented a decrease in chemical fertilizer and pesticide application rate of cooperative members. Moreover, Mojo et al. [6], Ma and Abdulai [5] found that membership in cooperatives shows an advantage in farm income.

Notwithstanding its important effect on reduction of environmental burdens, the results of aforementioned studies show that the assistance of cooperatives is heterogeneous regarding environmental and economic performance of farmers. Their results discriminated that age, gender, education level, farm size, experience in agricultural production, tenure security, human capital, accessibility to cooperatives and credit, and social networks significantly affect the environmental and economic performance of cooperative members [2, 5, 56–58]. While agricultural cooperatives in China have generally brought about environmental and economic co-benefits, more investigation will be needed to design policy instruments with the consideration of farmers’ heterogeneity to make cooperatives more meaningful and sustainable.

### Policy implications

The LCA results suggest that the main improvement strategies should be focused on the application and production of organic fertilizer, pesticides and fertilizers, and the results of CBA imply reducing the cost of labor power and chemical fertilizer. From this point of view, some useful technologies can reduce the environmental burdens while improving farm income for grape production. For instance, the indiscriminate use of organic and chemical fertilizers could be reduced through efforts to improve the efficiency of fertilizer use, such as soil testing and nutrient balance management [10, 50]. In addition, training courses should be available to inform vinegrowers about the proper amount and type of fertilizers and pesticides used for grape production [59]. Additionally, considering the shortage of organic fertilizer and its great contribution to the environmental impacts, the establishment of an integrated mixed crop-livestock system should be taken into consideration [18].

When the environmental performance of grape production is compared for the three organizational forms, the results show that IOF has the lowest environmental burdens, followed by FOC, and therefore, the highest environmental impacts were linked to SF. Meanwhile, the economic benefits in SF show an obvious decrease, ranging from 59.32% to 48.74% when compared to IOF and FOC, respectively. Taking the environmental and economic performance together, the total net benefits of IOF and FOC are higher than SF (72.1% and 58.86%, respectively). These results suggest that the conversion of SF into IOF or FOC may be an effective way to reduce the environmental impacts and increase the economic returns of grape production. However, to meet the requirements of a high level of skill and management or a better growing environment for high-quality grapes, the investment in vineyards of IOF and FOC was much higher than that of SF. However, the transaction costs, taken when contacting smallholder farmers, are very high [5]. In addition, as reported by Deng et al. [31] and Villanueva-Rey et al. [51], it will take at least 3 years of SF to convert into IOF or FOC, during which the yield of grapes is very low (according to the study of Ferrari et al. [49], during the conversion period, the grape would be produced with a productivity of 0, 30% and 70% in the first two, the third and the fourth year, respectively). Therefore, this approach should be further investigated with consideration of both the economic and environmental performance of IOF and FOC and the high cost and risk of conversion of SF into IOF or FOC.

Meanwhile, Vázquez-Rowe et al. [52] found that there is a difference in efficiency between different grape production sizes. From this point of view, increasing production size would reduce the environmental impacts and improve farm income of grape production. In China, the competition for land is increasing due to the great demand for land for the development of urban areas and industry and for increasing population [7]. This demand for land is a barrier to reducing environmental burdens and improving farm income by increasing production size. Therefore, a trade-off between improvement of economic and environmental performance of grape production and demand for land should be taken into consideration. Additionally, Ma and Abdulai [5] discerned that small-scale members of agricultural cooperatives were more beneficial than medium- and large-scale ones, implying that the proper grape production size should be further investigated.

Agricultural subsidies play an important role in supporting agricultural development and increasing farm income. Previous studies have proven that agricultural subsidies have a significant effect on the performance improvement of agro-food production [7]. The direct subsidies for grape production would encourage smallholder farmers to join the cooperative by increasing the expected profit and reducing the risk of conversion into IOF and FOC (such as the agricultural price support subsidies). However, indirect subsidies aimed at lowering the cost of materials and inputs would facilitate the conversion of SF into IOF and FOC. Therefore, the design of policy instruments to support grape production should be taken into consideration.

## Impact of the sampling approach

As mentioned above, though a stratified random sampling approach was used to select the vinegrowers who were interviewed, the vinegrowers of cooperatives and smallholder farmers may differ from each other, which will result in selection bias. The purpose of this study is to estimate the economic and environmental performance of cooperatives and smallholder farmers and compare the results of different production organizations aiming at providing some insights for vinegrowers, managers of cooperatives and policy makers. Although it may not affect our current results, the selection bias may result in the generalizability of our result being restricted to only Changli County and make our study fail to explain whether the cooperative is the driving force for the reduction of agricultural pollution. The results of our study demonstrate that the economic and environmental performance of grape cooperatives is better than the economic and environmental performance of smallholder farmers in Changli County; however, these results cannot explain where the better performance of cooperatives comes from since the vinegrowers of cooperatives we selected may differ from the smallholder farmers in many ways. Therefore, further study is needed to determine the driving force for agricultural pollution reduction.

## Conclusions and limitations

Agricultural cooperatives have been regarded as an important part of the modern agricultural production systems. This trend in China suggests the sustainable development of agricultural cooperatives. Considering increasing concerns about agricultural pollution, the environmental performance of agricultural cooperatives should be quantified. To the best of our knowledge, this study is the first to assess the environmental impacts of agricultural cooperatives and analyze their comparison with smallholder farmers. Taking grape production as an example, our study used LCA to quantify the environmental impacts, CBA to assess the economic performance of agricultural cooperatives and smallholder farmers and analyzed the comparison of LCA and CBA results between different organizational forms. And DEA was used to quantify the potential consequences of operational inefficiencies. The data were collected from 62 vinegrowers of Changli County, Hebei Province, China. Despite the necessity of further environmental improvement, the results show that the cooperatives have an overall effect on reducing environmental impacts and a higher economic return. Taking environmental and economic performance together, IOF and FOC presented a higher total net benefit of 985.79 and 1207.53 CNY/t than smallholder farmers. A significant economic saving can be achieved by improving the productivity of grape production.

This study provides a better understanding of the environmental and economic performance of agriculture cooperatives and smallholder farmers. The findings will help policy makers and vinegrowers in decision making to improve the performance of grape production in Changli County and promote the processes of regional development and Beijing-Tianjin-Hebei integration. However, there is nevertheless a set of limitations. First, fieldwork is conducted only in Changli County, and our attention is paid only to grape production. Thus, the findings may not be applicable to other regions or other types of industries. An investigation in other regions and other kinds of production systems should be carried out in future research. Second, the analysis in this study is performed with cross-sectional data, and only a stratified random sampling approach was used, leading to selection bias since the vinegrowers of cooperatives and smallholder farmers differ from each other in many ways. Further research should be conducted with panel data in which all important observed attributes are involved, and unobserved attributes are dealt with properly. Third, only descriptive statistics are used in our study, which may make the results less convincing regarding the environmental and economic performance of cooperatives. Prospective research may benefit from using some other quantitative methods, such as artificial intelligence and spatial analysis.

## Supporting information

S1 Table Title and source of links of the government reports. (PDF)

S1 File The questionnaire designed for field survey. (PDF)

S2 File Details on DEA method. (PDF)
