# Supplementary material for: Comparative analysis on environmental and economic performance of agricultural cooperatives and smallholder farmers: The case of grape production in Hebei, China
Source: PLoS One. 2021 Jan 25;16(1):e0245981. doi: 10.1371/journal.pone.0245981 (PMC7833222; doi:10.1371/journal.pone.0245981)
Supplement: S1 Table — (PDF) [file pone.0245981.s001.pdf]

S1 Table. Title and source of links of the government reports.

Title	Source of links
Report of 18th session of national congress of the communist party of China	http://cpc.people.com.cn/n/2012/1118/c64094-19612151.html
Report of 19th session of national congress of the communist party of China	http://cpc.people.com.cn/n1/2017/1028/c64094-29613660.html
Central Document No.1 2015	http://www.moa.gov.cn/ztzl/jj2020zyyhwj/yhwjhg/201603/t20160304_5039590.htm
Central Document No.1 2016	http://www.moa.gov.cn/ztzl/jj2020zyyhwj/yhwjhg/201701/t20170124_5465022.htm
Central Document No.1 2017	http://www.moa.gov.cn/ztzl/jj2020zyyhwj/yhwjhg/201802/t20180205_6136437.htm
Central Document No.1 2018	http://www.moa.gov.cn/ztzl/jj2020zyyhwj/yhwjhg/201902/t20190220_6172168.htm
Central Document No.1 2019	http://www.moa.gov.cn/ztzl/jj2020zyyhwj/yhwjhg/201902/t20190220_6336696.htm
Central Document No.1 2020	http://www.moa.gov.cn/ztzl/jj2020zyyhwj/2020zyyhwj/202002/t20200205_6336614.htm
