# Supplementary material for: Comparative analysis on environmental and economic performance of agricultural cooperatives and smallholder farmers: The case of grape production in Hebei, China
Source: PLoS One. 2021 Jan 25;16(1):e0245981. doi: 10.1371/journal.pone.0245981 (PMC7833222; doi:10.1371/journal.pone.0245981)
Supplement: S1 File — (PDF) [file pone.0245981.s002.pdf]

葡萄生产调查问卷

一、种植者信息

1.1 您的地址： ____

1.2 种植者类型： 企业种植基地☐　　农民专业合作社☐　　其他☐

1.3 您的年龄： ______________　　　　**您种植葡萄的年限：** ______________

1.4 您的学历： 小学☐　　中学☐　　大专☐　　本科☐　　硕士及以上☐

1.5 合作社名称： ____________________________

1.5 种植的主要葡萄品种、面积及用途

葡萄品种	种植面积（亩）	葡萄用途（鲜食、酿酒等）	葡萄品种	种植面积（亩）	葡萄用途（鲜食、酿酒等）
葡萄园总面积：__________亩					

二、种植葡萄所花费的成本

2.1 建园成本

葡萄园建立时间：		预计葡萄园可使用年限：　　年
如果是租用土地建葡萄园，葡萄园土地租金：　　元/亩/年		如果是自家土地建葡萄园，当地农用土地流转的费用一般是：　　元/亩/年
建园物质成本（元）		指建园时花费的所有物质费用，包括购买水泥、钢架、铁丝、农膜、基础灌溉设施等；
建园人工成本（元）		建园时花费的所有人工成本，包括平整土地、搭建葡萄架、挖排水沟、定植苗木等所有人工开支；
葡萄园每年新增维护费用：　　元/亩.年		

**修建葡萄园时，主要消耗的物质材料清单（包括葡萄苗木）**					
材料名称	数量[斤(升)等]	花费资金（元）	材料名称	数量[斤(升)等]	花费资金（元）

2.2 葡萄园每年的物质和人工成本（表格未列出的物质投入请填写在表格空白处）

**每年物质成本投入**					
项目	所需数量[斤(升)等/年]	花费资金(元/年)	项目	所需数量[斤(升)等/年]	花费资金(元/年)

**共计物质投入花费资金：__________元/年/亩**					

自有葡萄园农机具

机械名称	合计作业时间（小时）	耗油量（升/小时）	当地油价（元/升）	机械购置费用（元/台）	机械寿命（年）

租用葡萄园农机具

机械名称	合计作业时间（小时）	耗油量（升/小时）	当地油价（元/升）	机械租赁费用（元/台）	

每年雇佣人工（指领取工资、劳务费的人工）

生产全过程	用工数量（如果按月雇佣工人，请折合成以天为单位）（人·天）	雇佣人工花费总计（元）	备注
		元	

每年自家投入的人工（指不领取工资、劳务费用的人工）

生产全过程	折合花费在葡萄园的总计劳动时间（人·天）	当地平均工价（元/人/天）	备注

三、葡萄园的收益情况

葡萄品种	单产(斤/亩)	单价（元/斤）	葡萄品种	单产(斤/亩)	单价（元/斤）
您的整个葡萄园每年销售总收入是____________					

调查员姓名：　　　　调研时间：　　　　调研地点：

The questionnaire of grape production

1. information of vinegrower

1.1 your address: ____

1.2 the type of vinegrower: investor-owned-firms-led cooperatives☐ farmers-owned cooperatives☐ others☐

1.3 your age: ______________ **experience in grape cultivation (in years):** ______________

1.4 your education level: primary school☐ middle school☐ associate degree☐ bachelor degree☐ master or doctoral degree☐

1.5 the name of cooperative____________________________

1.5 the varieties, area and use of grape

Grape varieties	Area	Use (for fresh eat or wine et. Al.)	Grape varieties	Area	Use (for fresh eat or wine et. Al.)
Total hectare of vineyard: ________________					

2. the cost of grape production

2.1 cost of vineyard construction

When was your vineyard established:		How many years do your vineyard keep available:
If the land is rented, the rent is ________		If the land is your own, the profit of transfer of land rights is _____________
The cost of martials		Including all the martials cost
The cost of labor power		Including all the labor cost
maintenance cost of your vineyard every year:		

**Martials used to construct the vineyard (including the seedling)**					
Martial	Amount	Cost	Martial	Amount	Cost

2.2 cost of grape production every year

**Cost of Martials used for grape growing**					
Martial	Amount	Cost	Martial	Amount	Cost

Total cost:

Your own farm machinery

farm machinery	Total working hours	Fuel consumption	oil price	The price	mechanical life

The rent of farm machinery

farm machinery	Total working hours	Fuel consumption	oil price	The rent

Information about hiring workers

All stages of grape production	Total working days (man*day)	The cost	Note

Information about family labors

All stages of grape production	Total working days (man*day)	Local average cost of labor power	Note

3. the return of vineyard

Grape varieties	Yield	Price	Grape varieties	Yield	Price

Total gross return_____________

Investigator: **Investigation time:** **Investigation place:**
