# Supplementary material for: Comparative analysis on environmental and economic performance of agricultural cooperatives and smallholder farmers: The case of grape production in Hebei, China
Source: PLoS One. 2021 Jan 25;16(1):e0245981. doi: 10.1371/journal.pone.0245981 (PMC7833222; doi:10.1371/journal.pone.0245981)
Supplement: S2 File — (PDF) [file pone.0245981.s003.pdf]

Details on DEA method

The input of diesel, water, chemical fertilizers (including N, P, K), organic fertilizer, pesticides, electricity, plastic film and labor power are selected as the DEA inputs, whereas the yield of grapes is the unique DEA output. All the selected operational inputs for DEA implementation are assumed to be independent from each other. Therefore, the land used for vineyard is not included since its minimization would affect other inputs. Similarly, direct emissions from the application of operational inputs are also not included in the DEA matrix, because of their direct proportion to the DEA inputs.

The selected DEA model is input-oriented slacks-based measure of efficiency (SBM-I) model with constant returns to scale (CRS). The selection of SBM is based on its advantage in flexibility since this model compute the efficiency of decision making units (DMU) without considering the units of measure used for the different inputs/outputs. On the other hand, the selection of an input-oriented model is based on the fact that our purpose is to minimize the use of operational inputs to reduce the environmental impacts of grape production without reduction of yield level. Additionally, we assume the interviewed vinegrowers operate in a competitive market and they work under the same incentive and competitiveness conditions, which leads to the selection of CRS.

DEA-solver Pro is used for DEA computation. The results are tabulated in Table S2.1, of which No.1-12 represent vinegrowers of IOF, No.13-42 represent vinegrowers of FOC and No.43-62 represent vinegrowers of SF.

Table S2.1. SBM resutls

No.	Score	No.	Score	No.	Score	No.	Score
1	1	17	0.5344	33	0.5977	49	0.4162
2	1	18	0.6540	34	0.3619	50	0.3913
3	0.5815	19	0.6732	35	0.3577	51	0.4397
4	1	20	0.7619	36	0.4430	52	0.8924
5	0.7965	21	1	37	0.3360	53	1
6	1	22	1	38	0.6175	54	0.7346
7	0.5933	23	0.5682	39	0.5485	55	0.7619
8	0.6266	24	1	40	1	56	0.3237
9	0.7716	25	1	41	0.4614	57	0.4189
10	1	26	1	42	0.3677	58	1
11	1	27	0.4984	43	1	59	0.7530
12	1	28	1	44	0.5862	60	1
13	0.8244	29	0.4324	45	0.3831	61	1
14	1	30	0.5114	46	1	62	0.4685
15	0.6329	31	0.5246	47	1		
16	0.4756	32	1	48	0.3811		

Given the fact that there are 23 vinegrowers being deemed efficient, we use a super-efficiency analysis to find out which vinegrower performs the best. Similarly, the super-SBM-I with CRS is selected as the DEA model. The results are listed in Table S2.2.

Table S2.2. super-SBM resutls

No.	Score	No.	Score	No.	Score	No.	Score
1	1.0326	12	1.0077	26	1.0241	47	1.0753

2	1.1539	14	1.0059	28	1.0004	53	1.1082
4	1.3378	21	1.0646	32	1.0695	58	1.1268
6	1.0572	22	1.0117	40	1.0633	60	1.1575
10	1.0584	24	1.1280	43	1.2147	61	1.0956
11	1.0252	25	1.0906	46	1.0290		

The DEA results are combined with LCA and CBA results by following a five-step LCA+DEA method (see more details in Vázquez-Rowe (2012)).

Reference

Vázquez-Rowe I, Villanueva-Rey P, Iribarren D, Teresa Moreira M, Feijoo G. Joint Life Cycle Assessment and Data Envelopment Analysis of Grape Production for Vinification in the Rías Baixas Appellation (NW Spain). Journal of Cleaner Production. 2012;27: 92-102.
